# The association of polytherapy and psychiatric comorbidity in epilepsy

**DOI:** 10.1016/j.yebeh.2024.110215

**Published:** 2025-02

**Authors:** Mercy A. Odhiambo, Gilbert K. Kaingu, Maria Mumbo, Karin Kipper, Josemir W. Sander, Charles R.J.C. Newton, Symon M. Kariuki, on behalf of the EPInA investigators

**Affiliations:** a Neurosciences Unit, KEMRI Wellcome Trust Research Programme, P.O. Box 230-80108, Kilifi, Kenya; b The Open University, P.O. Box 197, Milton Keynes, MK7 6BJ, United Kingdom; e UCL Queen Square Institute of Neurology, London WC1N 3BG and Chalfont Centre for Epilepsy, Chalfont St Peter, SL9 0RJ, United Kingdom; f Department of Neurology, West China Hospital, Sichuan University, Chengdu 610041, China; c Department of Public Health, School of Human and Health Sciences, Pwani University, P.O Box 195-80108, Kilifi, Kenya; d Department of Psychiatry, University of Oxford, Oxford, United Kingdom; g African Population and Health Research Centre, Nairobi, Kenya

**Keywords:** Mental health problems, Community epilepsy clinic, Anti-seizure medications, Polytherapy, Africa

## Abstract

**Purpose:**

Managing epilepsy may require using more than one anti-seizure medication (ASM). While combination therapy may help, risks, including psychiatric problems, are not fully explored in Africa. We examined the relationship between polytherapy and psychiatric comorbidities among attendees of an epilepsy community clinic.

**Methods:**

We prospectively assessed individuals attending an outpatient clinic in Kilifi, Kenya, for patterns of ASM prescribing (mono- or polytherapy) and reviewed psychiatric diagnoses. We used the Psychosis Screening Questionnaire and the Patient Health Questionnaire Version 9 to assess for psychosis and depression, and the Child Behavior Checklist to assess for emotional and behavioural problems. We conducted a cross-sectional logistic regression analysis to determine factors associated with polytherapy and examine the impact of polytherapy and specific medication on psychiatric comorbidities.

**Results:**

Of 3,016 attendees, most were on older ASM (99.7 %), with about a third (32.9 %) on polytherapy. The most commonly co-administered drugs were phenobarbital and carbamazepine (13.0 %). Children were less likely to be on multiple medications than adults, and there was no difference between the sexes. Polytherapy was associated with focal to bilateralised seizures (aOR 1.2 [95 % confidence interval:1.0–1.4]) and frequent seizures (aOR = 2.1 [1.5–2.9]). Combining drugs increased the likelihood of any psychiatric problems (aOR = 1.3 [1.0–1.8]), with polytherapy associated with depression (aOR = 2.9 [1.0–8.4]) and psychosis (aOR = 1.9 (1.0–3.6)).

**Conclusion:**

Polytherapy, especially with older drugs, is associated with psychiatric comorbidities in this population. Resorting to polytherapy needs to be carefully considered. Prioritizing research into the long-term effects of ASM on psychiatric comorbidities is crucial for improving mental health outcomes in epilepsy, particularly in low-income settings.

## Highlights

• Combinations of anti-seizure medications (ASM) are common practice in people with epilepsy.
• Risks for polytherapy, including psychiatric problems, have not been fully explored in Africa.
• ASM combination in epilepsy may be associated with mental health comorbidity.
• Longitudinal studies may clarify the relationship between polytherapy and mental health comorbidity.
• Capacity building among healthcare workers on appropriate ASM combinations may be helpful.

## 1 Introduction

A combination of anti-seizure medications (ASM) or polytherapy is often used by people with epilepsy who respond poorly to treatment with a single ASM[1]. Polytherapy’s goal is to achieve synergistic therapeutic effects, resulting in better seizure control or minimizing toxicity by allowing using lower individual doses[2]. In people on multiple ASM, trends towards greater seizure freedom and treatment retention at 12 months were seen compared to monotherapy, although not statistically significant[3]. If properly selected, polytherapy may be as effective and not necessarily more toxic than monotherapy[4].

The criteria for combination therapy in epilepsy have been proposed. Combining ASM with complementary mechanisms of action (e.g. those acting on sodium channels combined with GABA agonists) is recommended[5]. Due to multiple ASM action mechanisms, any combination will likely be synergistic[6]. The prescriber’s choice of drug therapy may be influenced by introducing new drugs or, in some countries, by funding mechanisms such as clinical trials or reimbursement schemes. For instance, in resource-limited settings, people with epilepsy are likely to use a combination of including phenobarbital, phenytoin or carbamazepine. These strong enzyme inducers are affordable and readily available despite an increased risk of adverse drug reactions (ADR) including intellectual disability, emotional and behavioral problems in children[7] and neuropsychiatric symptoms[8].

Switching of ASM, especially when in remission, can be complex and sometimes not advisable, as there is a risk of seizure recurrences. Drug-drug interactions may also lead to altered levels. For instance, to avoid toxicity, combining sodium valproate with lamotrigine usually requires reducing lamotrigine dosage given the enzyme-inhibiting properties of valproate. Unfortunately, clinicians often focus more on seizure control and may overlook ADRs.

There is some evidence of polytherapy of older ASM, i.e. phenobarbital, phenytoin and carbamazepine, in high-income countries (HIC), often using pragmatic clinical studies[3], [9]. A knowledge gap exists, however, in low- and middle-income countries (LMIC) where these ASMs are commonly prescribed. Studies have also shown increased treatment effectiveness based on seizure control by adding lamotrigine to older ASMs, but lamotrigine is not readily available in LMIC. Some Asian studies identified a long history of epilepsy (i.e., ⩾five years), frequent seizures (i.e., > two seizures), symptomatic epilepsy, and multiple seizure types as polytherapy-associated factors [10]. Some of these factors may be important in Africa, but have not been investigated.

ADR associated with mono- or polytherapy, including psychiatric problems, are documented [11] but have not been systematically investigated in Africa. There is emerging evidence that some ASMs may be associated with an increased risk of psychiatric problems, especially psychosis. [12] This may be difficult to distinguish from the manifestation of epilepsy, such as brief ictally-related psychotic symptoms. This distinction may not be possible in a single study. Still, exploratory studies aimed at establishing associations between ASM polytherapy and psychiatric symptoms accounting for various proxies of the disease process may provide preliminary evidence. This may help inform ASM prescription in special groups such as pregnant women and advocate for investment in newer ASM by governments in resource-limited settings, including Africa.

Within the Kilifi Health and Demographic Surveillance System (KHDSS), the burden of epilepsy has been previously estimated at 20–41/1,000[13]. The epilepsy treatment gap in Kilifi was high based on surveys conducted between 2008 and 2011, estimating this at 80 % based on adherence and the presence of optimal blood levels of ASM[14]. Mental health problems are common in this population, with up to 11 % of children experiencing behavioural or emotional issues[15]. In adults, only a proportion with mental health problems visit outpatient facilities in the area, with many living with depression in the community undiagnosed[16].

We described ASM prescription patterns at an outpatient epilepsy clinic in Kilifi, Kenya. We hypothesized that clinical judgment would influence prescription patterns rather than an explicit criterion for drug choice, such as complementary mechanisms of action. We also aimed to determine individual and clinical factors associated with polytherapy and examine the association of ASM and polytherapy use with psychiatric symptoms. We hope these findings will form the basis for prospective follow-up of treatment naïve people. This will allow the longitudinal observation of arising psychiatric symptoms following a change of ASM or due to deterioration of the condition.

## 2 Materials and methods

### 2.1 General methodology

The study setting was was the epilepsy and neurodevelopmental clinic in Kilifi. It is run jointly by Kilifi County Hospital and the KEMRI Wellcome Trust Research Programme (KWTRP) on the coast of Kenya. The clinic serves residents of a defined area, i.e. the KHDSS, and attendees residing in the greater Kilifi County and coastal region of Kenya. The KHDSS covers an estimated area of 891  km^2^ with over 280,000 residents[17]. The residents are Mijikenda, a Bantu grouping of nine ethnic groups, with Giriama (45 %), Chonyi (33 %) and Kauma (11 %) dominating. This population’s literacy levels are low, estimated at only 45 %. For this analysis, children and adults with a diagnosis of epilepsy who had attended the clinic at least twice during the study period and were on at least one ASM were included. The exclusion criteria were children and adults who did not have a diagnosis of epilepsy, who attended the clinic for the first time or who were not on any ASM.

We documented details of each visit to the clinic in online questionnaires hosted in the electronic data capture system REDCap®. Electroencephalography (EEG) recordings were obtained based on clinical indications. We classified abnormal EEG for any recording that showed evidence of an abnormal background, focal changes, interictal epileptiform activity or an abnormal response to either of the activation procedures (hyperventilation and photic stimulation) as previously described[1]. Clinicians systematically administered questionnaires related to psychiatric comorbidities among people attending the clinic. Socio-demographic information, including age, sex, residence, seizure semiology and frequency, ASM prescribed, EEG data and history, including duration and psychiatric symptoms, were collected. Epilepsy was defined as a history of two unprovoked seizures occurring 24 h apart. Seizure semiology was initially classified according to the International League Against Epilepsy (ILAE) criteria based on onset as either generalized or focal with further stratification into focal to bilateral, tonic, atonic, myoclonic, absence and focal impaired awareness seizures. Seizure frequency was stratified into three: daily, weekly, and monthly seizures.

Psychiatric problems were documented in several ways. Firstly, participants were asked if they had received a diagnosis of any psychiatric problem, psychosis, or depression at any point. This was documented as self-reported psychosis. Secondly, the Psychosis Screening Questionnaire (PSQ) was used to assess lifetime psychosis or psychosis in the last year. The PSQ probes five domains (hypomania, thought insertion, paranoia, strange experiences, and hallucinations). An affirmative reply in any domain is considered a positive diagnosis. Depression was assessed using the Patient Health Questionnaire Version 9 (PHQ-9). The PHQ-9 consists of nine questions about loss of interest, feelings of depression, sleep, appetite problems and suicidal tendencies on a four-point Likert scale, with more intense depressive feelings getting higher scores. Emotional and behavioural problems in children were assessed using the Child Behavior Checklist (CBCL), which consists of 113 questions on externalizing and internalizing issues scored on a three-point Likert scale, with more problematic behaviours scoring higher. During analysis, we assessed the psychiatric diagnoses as any self-reported psychiatric problem, psychosis (whether self-reported or assessed using PSQ), depression (whether self-reported or assessed using PHQ-9), behavioural problems in children (assessed using the CBCL) and combined mental health problems (a sum of participants with any psychiatric problem, psychosis or depression). Monotherapy was defined as individuals taking only one of these ASMs: phenobarbital, carbamazepine or sodium valproate, phenytoin to manage their seizures, while polytherapy was defined as taking more than one ASM. Most clinic attendees were prescribed these ASMs, so our analysis focused on these four ASMs.

### 2.2 Ethical considerations

Clinical and mental health data for this analysis was collected as part of routine care aimed at optimizing epilepsy treatment and psychiatric comorbidities. Verbal consent was obtained during routine clinic appointments, and written informed consent was obtained only when blood samples were collected. An overall study protocol covering the epilepsy clinic was reviewed and approved by the Kenya Medical Research Institute Scientific Ethics Review Unit (KEMRI/SERU/CGMR-C/125/3701)**.**

### 2.3 Statistical analysis

We used Stata Version 17 (StataCorp. 2021. *Stata Statistical Software: Release 17*. College Station, TX: StataCorp LLC) for the analysis. Descriptive population statistics and prescription patterns were computed as percentages, and differences in monotherapy versus polytherapy use were compared using Pearson’s chi-squared test. We used a Venn diagram to display prescription patterns for the commonly used ASM at the clinic. To investigate the association of polytherapy with various socio-demographic factors, seizure semiology and epilepsy, we conducted univariable and adjusted logistic and linear regression models. We adjusted for age, sex, and residence in the univariable regression. Similar analyses were conducted to examine the association between polytherapy and specific ASM use and psychiatric symptoms. Interaction terms were added to the adjusted logistic regression, probing and exploring the relationship between polytherapy and psychiatric symptoms. This also further accounted for specific factors such as being a child, seizure type and frequency, EEG results and duration of epilepsy. Lastly, we conducted a sensitivity analysis to explore the factors associated with experiencing psychiatric symptoms among people on polytherapy.

## 3 Results

### 3.1 General description

From 16th March 2019 to 6th May 2024, 3016 people attended the clinic at least twice and were included in this analysis. One-thousand-seven hundred and ninety-five (59.5 %) of the attendees were children, 1682 (56.1 %) were males, and over half resided within the KHDSS (Table 1). There were more children than adults (59.5 % vs 40.4 %; p > 0.001) and more females than males (55.7 % vs 44.2 %; p = 0.018) attending the epilepsy clinic. Slightly less than half of these people had focal seizures, which were more common in adults than children (57.8 % vs 42.2 %; p < 0.001).

**Table 1:** Description of Attendees of the Epilepsy Clinic in Kilifi, Kenya.

**Variable**	**Monotherapy N = 2023 (67.1 %)**	**Polytherapy N = 993 (32.9 %)**	**Total N = 3016**	**P value**
**Socio-demographic factors**				
Median age	11.0(4.0–23.0)	18.0 (6.0–29.0)	13.0(5.0–25.0)	**<0.001**
Children	1303 (64.4)	492 (49.5)	1795 (59.5)	**<0.001**
Sex (male)	1138 (56.2)	544 (54.7)	1682 (55.7)	0.445
Residence in KHDSS	926/1853 (49.9)	358/927 (38.6)	1284/2780 (46.2)	**<0.001**

**Seizure types**				
Focal	790/1982 (39.8)	405/969 (41.8)	1195/2951 (40.5)	0.314
Generalized	1192/1982 (60.1)	564/969 (58.2)	1756/2951 (59.5)	0.314

**Specific types**				
Focal to bilateral	507/1982 (25.5)	290/969 (29.9)	797/2951 (27.0)	**0.012**
Tonic	195/1982 (9.8)	101/969 (10.4)	296/2951 (10.0)	0.620
Atonic	33/1982 (1.6)	14/969 (1.4)	47/2951 (1.6)	0.654
Myoclonic	133/1982 (6.7)	107/969 (11.0)	240/2951 (8.1)	**<0.001**
Absence	61/1982 (3.1)	22/969 (2.3)	83/2951 (2.8)	0.213
Complex partial	103/1982 (5.2)	54/969 (5.6)	157/2951 (5.3)	0.669
Other focal	190/1982 (9.6)	66/969 (6.8)	256/2951 (8.6)	**0.012**
Other seizure types	21/1982 (1.1)	4/969 (0.4)	25/2951 (0.8)	0.072

**Seizure frequency**				
Daily seizures	106/716 (14.8)	93/409 (22.7)	199/1125 (17.7)	**0.001**
Weekly seizures	131/711 (18.4)	101/402 (25.1)	232/1113 (20.8)	**0.008**
Monthly seizures	362/812 (44.5)	256/443 (57.8)	618/1255 (49.2)	**<0.001**

**Anti-seizure medications**				
Phenobarbital	584 (28.9)	723 (72.8)	1307 (38.26)	**<0.001**
Carbamazepine	847 (41.8)	657 (66.2)	1504 (49.8)	**<0.001**
Sodium valproate	551 (27.2)	558 (56.2)	1109 (36.7)	**<0.001**
Phenytoin	36 (1.8)	47 (4.7)	83 (2.7)	**<0.001**
First generation ASM	1967/1976 (99.5)	987/987 (100.0)	2954/2863 (99.7)	**<0.034**

**Epilepsy factors**				
Abnormal EEG	378/526 (71.8)	119/149 (79.8)	497/675 (73.6)	**0.050**
Median duration of epilepsy (IQR)	4.0 (1.0–10.0)	10.0 (4.0–20.0)	6.0 (2.0–15.0)	**<0.001**
Status epilepticus (>5min)	713/906 (78.7)	347/454 (76.4)	1060/1360 (77.9)	0.342
Status epilepticus (>30 min)	248/906 (27.3)	117/454 (25.7)	365/1360 (26.8)	0.529

**Psychiatric comorbidity**				
Any psychiatric problems	196/723 (27.1)	112/368 (30.4)	308/1091 (28.2)	0.249
Combined psychosis (self-reported and PSQ)	32/222 (14.4)	31/112 (27.6)	63/334 (18.9)	**0.003**
Combined depression (self-reported and PHQ9)	7/736 (0.9)	11/380 (2.9)	18/1116 (1.6)	**0.015**
Emotional and behavioural problems in children (CBCL)	102/175 (58.3)	39/45 (86.7)	141/220 (64.1)	**<0.001**

CBCL = Child Behavior Checklist; PHQ = Patient health Questionnaire; EEG = electroencephalophy; ASM = anti-seizure medication; IQR = interquartile range.

### 3.2 Prescription pattern

**Fig. 1:**
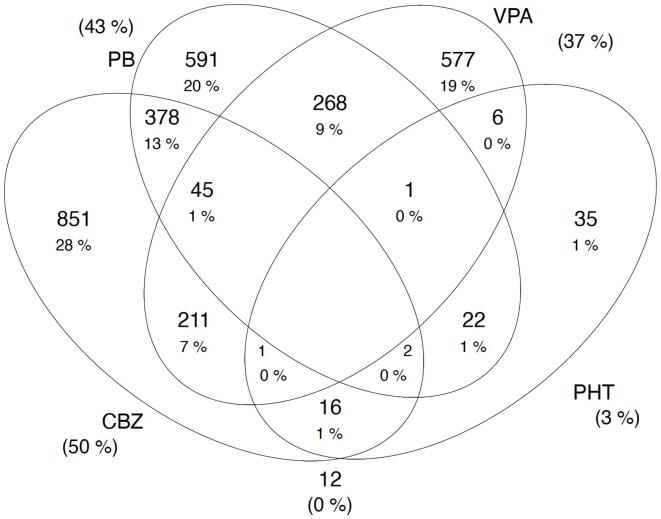
Prescription pattern of anti-seizure medications.

Most (3,093 (99.6 %)) attendees were on older ASM, with carbamazepine (50.0 %) being the most common, followed by phenobarbital (43.0 %) (Fig. 1). ASM polytherapy was prescribed for 993 (32.9 %) people. The most common ASM combination was phenobarbital and carbamazepine (13.0 %), followed by phenobarbital with sodium valproate (9.0 %). Polytherapy was more common in adults than children (50.4 % vs 49.5 %; p < 0.001), in focal to bilateralised seizures (29.9 %), and in people with psychiatric symptoms than those without (27.6 % vs 14.4 %; p = 0.003), among other characteristics (Table 1).

**Fig. 2:**
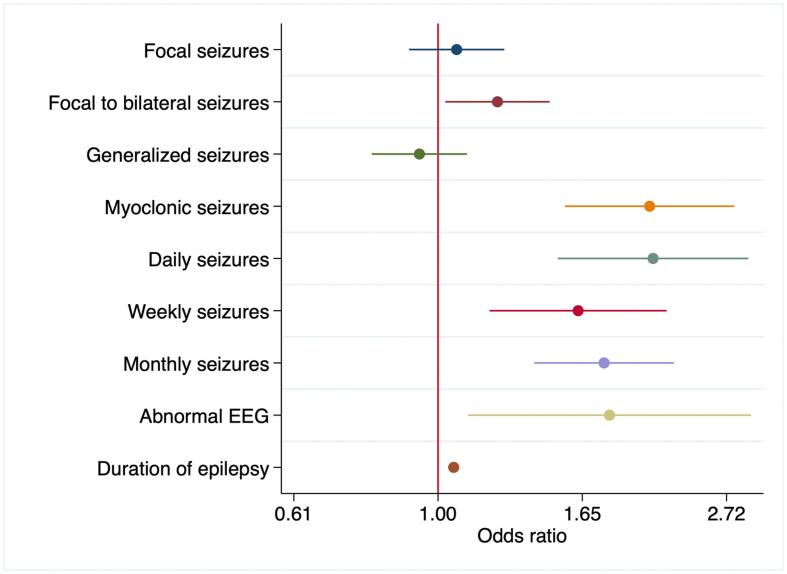
Features associated with polytherapy.

Several factors were associated with polytherapy in the univariable analysis, with being a child (odds ratio (OR) = 0.54 (95 % confidence interval (CI): 0.46–0.63); p < 0.001), focal to bilateralised seizures (OR = 1.24 (95 %CI:1.04–1.47); p < 0.001) and epilepsy duration (OR = 1.05 (95 %CI:1.04–1.07); p < 0.001) (Table 2). The findings were similar in the adjusted analysis, with polytherapy being strongly associated with focal to bilateralised seizures (aOR = 1.24 (95 %CI:11.04–1.49); p = 0.015), daily/frequent seizures (aOR = 2.08 (95 %CI:1.49–2.89); p < 0.001) and epilepsy duration (aOR = 1.06 (95 %CI:1.04–1.07); p < 0.001) (Fig. 2).

**Table 2:** Factors Associated with Polytherapy (Univariable and Adjusted Analysis: sex, being a child, residence).

**Variable**	**Univariable analysis**			**Adjusted Analysis**	
	**Odds Ratio (95 % CI)**	**P value**	**Odds Ratio (95 %CI)**		**P value**
**Socio-demographic factors**					
Child	0.54 (0.46–0.63)	**<0.001**		**−**	**−**
Sex (males)	0.94 (0.80–1.09)	0.445		−	−
Residence in KHDSS	0.62 (0.53–0.73)	**<0.001**		−	−

**Seizure types**					
Focal	1.08 (0.92–1.26)	0.314		1.07 (0.91–1.27)	0.367
Generalized	0.92 (0.78–1.07)	0.314		0.92 (0.78–1.09)	0.367

**Specific types**					
Focal to bilateral	1.24 (1.04–1.47)	**0.013**		1.24 (1.04–1.49)	**0.015**
Tonic	1.06 (0.82–1.37)	0.620		1.12 (0.86–1.47)	0.376
Atonic	0.86 (0.46–1.62)	0.654		1.022 (0.53–1.96)	0.933
Myoclonic	1.72 (1.32–2.25)	**<0.001**		2.04 (1.52–2.74)	**<0.001**
Absence	0.73 (0.44–1.19)	0.215		0.83 (0.50–1.40)	0.507
Complex partial	1.07 (0.76–1.51)	0.659		1.10 (0.77–1.57)	0.581

**Seizure frequency**					
Daily seizures	1.69 (1.24–2.30)	**0.001**		2.08 (1.49–2.89)	**<0.001**
Weekly seizures	1.48 (1.10–1.99)	**0.008**		1.60 (1.18–2.17)	**0.002**
Monthly seizures	1.70 (1.34–2.15)	**<0.001**		1.78 (1.39–2.26)	**<0.001**

**Epilepsy factors**					
Abnormal EEG	1.05 (1.04–2.41)	0.052		1.76 (1.08–2.88)	**0.023**
Duration of epilepsy	1.05 (1.04–1.07)	**<0.001**		1.06 (1.04–1.07)	**<0.001**
Status epilepticus (>5min)	0.87 (0.67–1.14)	0.342		0.80 (0.61–1.06)	0.128
Status epilepticus (>30 min)	0.92 (0.71–1.18)	0.530		0.94 (0.72–1.22)	0.653

CI = Confidence intervals; EEG = electroencephalography; KHDSS = Kilifi Health and Demographic Surveillance System.

### 3.3 Association of polytherapy with psychiatric comorbidities

In the adjusted analysis, polytherapy increased the likelihood of experiencing psychiatric problems (aOR = 1.37 [1.02–1.84]; p = 0.035), psychosis (aOR = 1.98 [1.08–3.62]; p = 0.025), depression (aOR = 2.92 [1.01–8.40]; p = 0.046) and all reported mental health problems (aOR = 1.42 [1.08–1.87]; p = 0.012) (Table 3); all assessed with standardized scales. These associations were similar to analysis for reported psychosis (OR = 2.27 [1.30–3.97]; p = 0.004) and depression (OR = 3.10 [1.19–8.07]; p = 0.020) (supplementary Table 1). In the sensitivity analyses, children on polytherapy with focal to bilateral motor seizure types were more likely to have behavioural problems (ß coefficient = 1.52 [0.09–2.95; p = 0.037]) while individuals on polytherapy with a longer duration of illness were more likely to report psychosis (aOR = 1.08 [1.01–1.16]; p = 0.012) (supplementary Table 3).

**Table 3:** Association between Polytherapy and Specific Anti-seizure Medications with Psychiatric Comorbidities (Adjusted Analysis).

**Variable**	**Any psychiatric problems**		**Psychosis**		**Depression**		**Combined psychiatric problems**		**Behavioural problems**	
	**aOR (CI)**	**P value**	**aOR (CI)**	**P value**	**aOR (CI)**	**P value**	**aOR (CI)**	**P value**	**ß Coef.(CI)**	**P value**
Polytherapy	1.37 (1.02–1.85)	**0.035**	1.98 (1.08–3.62)	**0.025**	2.92 (1.01–8.40)	**0.046**	1.42 (1.08–1.87)	**0.012**	0.16 (−0.27–0.59)	0.463
Carbamazepine	1.73 (1.17–2.56)	**0.005**	1.59 (0.67–3.80)	0.289	1.78 (0.30–10.49)	0.520	1.64 (1.14–2.36)	**0.007**	0.08 (−0.40–0.58)	0.722
Sodium Valproate	1.42 (0.97–2.08)	0.068	0.31 (0.08–1.13)	0.078	3.35 (0.47–23.89)	0.227	1.31 (0.91–1,90)	0.138	0.50 (−0.17–1.02)	0.058
Phenobarbital	0.35 (0.22–0.55)	**<0.001**	0.65 (0.22–1.88)	0.429	Omitted**	−	0.37 (0.24–0.57)	**0.001**	−0.82 (−1.39-(−0.25)	**0.005**
Phenytoin	1.18 (0.29–4.81)	0.810	4.54 (0.90–22.87)	0.066	5.98 (1.05–33.75)	**0.043**	2.53 (0.85–7.46)	0.092	0.16 (−2.44–2.78)	0.896

aOR = adjusted odds ratio; ß Coef = beta coefficient; CI = Confidence intervals.

** Omitted due to collinearity.

### 3.4 Association of ASM and psychosis symptoms

Individually, phenobarbital appeared to reduce the risk of psychiatric (aOR = 0.35 (95 %CI:0.22–0.55); p < 0.001) and behavioural problems in children (ß coefficient = -0.82 (95 %CI:-1.39-(−)0.25); p = 0.005). Carbamazepine seemed to increase the likelihood of reported psychiatric problems (aOR = 1.73 [1.17–2.56]; p = 0.005), and all reported mental health problems (aOR = 1.64 [1.14–2.36]). Individuals on phenytoin were almost six times more likely to report depression (aOR = 5.98[1.05–33.75]; p = 0.04) (Table 3).

## 4 Discussion

We estimated the pattern of ASM use, the associated factors, and the impact on reported psychiatric symptoms. Older ASM were commonly prescribed in this setting, especially carbamazepine and phenobarbital. Carbamazepine was the most widely prescribed drug, and this could be attributed to the high prevalence of focal epilepsies triggered by symptomatic causes, including infections[18]. Phenobarbital, the second most commonly prescribed ASM, was frequently used in combination with carbamazepine. Similar to a Bengali study[19], we found that dual ASM therapy was common, while more than two ASM combinations were uncommon.

About a third of clinic attendees were on polytherapy, similar to the proportion reported in South Africa[20], but slightly less than in India[21]. A large proportion of drug-responsive epilepsy, the long duration of illness and access to newer ASMs such as levetiracetam and topiramate not available in Kilifi may influence the higher use of polytherapy in India[21]. In poor settings, it is challenging to define refractory epilepsy because of limited access to newer ASM. For instance, the observed status epilepticus (23 %) or daily/frequent seizures (17 %) would be termed drug-resistant epilepsy and may require combination therapy if the newer drugs were available.

We found that polytherapy use was influenced by epilepsy duration, seizure type, and seizure frequency. People with a longer duration of epilepsy were more likely to be on polytherapy, as was reported in China[22]. However, unlike our study, they included people in two-year seizure remission. Regarding seizure types, polytherapy was common in people with focal seizures, which would be expected in an area where symptomatic epilepsy is common[18]. Symptomatic epilepsy and its associated comorbidities, including intellectual disability, may increase the risk for refractory epilepsy[23] and the need for polytherapy. Seizure frequency has been used as the endpoint for most clinical trials evaluating the effectiveness of ASM[24], and it is not surprising that polytherapy use was common in those with frequent seizures. Similar to our findings, another Indian study[25], found similar associations between polytherapy and seizure frequency, epilepsy duration, and multiple seizure types, including focal seizures. Notably, people with dissociative seizures may be placed on polytherapy when presumed to have refractory epilepsy, but we did not explore this, and evidence is lacking.

Psychiatric symptoms among people with epilepsy were associated with ASM polytherapy, including an increased risk for all mental health problems as well as depression and psychosis. Similarly, a recent African review of psychiatric comorbidities in epilepsy studies found depressive symptoms to be associated with polytherapy[26]. Many of these studies had small sample sizes, and the heterogeneity of the included studies may have influenced the findings. Other studies from HIC have also reported an increased risk for psychiatric and behavioural problems following the use of various ASMs[12].

The risk of psychiatric symptoms was reduced with the use of phenobarbital, perhaps because it is commonly used in generalized seizures whose outcomes may be favourable. This finding was similar to a Chinese study which showed some improvement in neuropsychological and cognitive outcomes among people with epilepsy taking phenobarbital[27]. An Indian study found that phenobarbital was not associated with behavioural problems in children[28], unlike what had been previously postulated[29]. Conversely, carbamazepine (CBZ) was associated with psychiatric problems, explained by several reasons. The emergence of emotional issues and psychosis-like symptoms following the initiation of carbamazepine, which cleared following discontinuation, has been documented in case reports [30]. Carbamazepine may have been prescribed for mood disorders[31], which are common comorbidities of epilepsy, and for focal epilepsy, which are often associated with mental health problems[32]. However, other studies from HICs in America[12] and Japan reported fewer psychiatric and behavioural side effects with CBZ use[33]. Individuals on phenytoin (PHT) had a higher likelihood of experiencing psychiatric problems, especially depression and this has been previously reported in several case studies, including some from India[34] one of which reported psychosis associated with PHT toxicity[35]. However, another review reported that PHT was likely to cause behavioural problems and affect cognition with fewer effects on mood[36]. Accumulating evidence suggests these mood disorders are due to phenytoin toxicity from elevated levels[34], but underlying brain damage should be ruled out.

We also noted that it might be difficult to distinguish between psychiatric symptoms related to the use of ASM and interictal seizure activity. This underlines the significance of understanding the relationship between combination therapy and psychiatric and behavioural problems.

## 5 Strengths and limitations

This analysis is based on a large dataset of clinic visits accumulated over a long duration and is well-powered to determine associations. Psychiatric symptoms were assessed using standardized scales adapted for the local population. The study setting is a rural population where rates of migration and immigration are low, and the results are generalizable to populations living along the Kenyan coast. As a cross-sectional analysis, it is not possible to establish causality and longitudinal follow-ups are needed. Data on ASM use are based on documented clinical assessments and individual self-reports. They may be unreliable in situations where drug levels are discordant with the dosages taken because of biological reasons or non-adherence. We also did not assess the proportion of people with dissociative seizures, some of whom could be on polytherapy. Lastly, several other ADRs were not available for analysis.

## 6 Conclusion

Epilepsy management with ASM polytherapy was associated with psychiatric comorbidities in our population. Initiation of polytherapy should be carefully considered, and more work on the longitudinal impact of ASM on psychiatric comorbidities should be prioritized to clarify their role in influencing mental health. Capacity building among healthcare workers on appropriate ASM combinations may be helpful.

## 7 Data sharing

We welcome collaborations. The data used in this study are part of the EPInA Project, which is underway in Kenya, Tanzania, and Ghana. The data collected for this study will be made available in keeping with the KWTRP’s Data Governance Policy. For requests, email the KWTRP’s Data Governance Committee at dgc@kemri-wellcome.org.

## CRediT authorship contribution statement

**Mercy A. Odhiambo:** Writing – review & editing, Writing – original draft, Visualization, Project administration, Methodology, Formal analysis, Data curation. **Gilbert K. Kaingu:** Writing – review & editing, Writing – original draft, Investigation, Data curation. **Maria Mumbo:** Writing – review & editing, Validation, Methodology, Investigation. **Karin Kipper:** Writing – review & editing, Writing – original draft, Validation, Supervision, Methodology. **Josemir W. Sander:** Writing – review & editing, Writing – original draft, Validation, Supervision, Methodology, Funding acquisition, Formal analysis, Conceptualization. **Charles R.J.C. Newton:** Writing – review & editing, Writing – original draft, Validation, Supervision, Methodology, Funding acquisition, Formal analysis, Data curation, Conceptualization. **Symon M. Kariuki:** Writing – review & editing, Writing – original draft, Supervision, Methodology, Conceptualization.

## Funding

This work was supported by the National Institutes of Healthcare and Research [grant number NIHR200134].

## Declaration of competing interest

The authors declare the following financial interests/personal relationships which may be considered as potential competing interests: JWS reports personal fees and research grants from Eisai, UCB, and Angelini Pharma outside the submitted work. Other authors do not have conflicts of interest to declare..
